# Parental traditional medicine use for children and associated factors in Harar City, Harari Regional State, Eastern Ethiopia: a community-based cross-sectional study

**DOI:** 10.3389/fped.2025.1546455

**Published:** 2025-06-09

**Authors:** Ayichew Alemu, Fentahun Meseret, Mulualem Keneni, Fenta Wondimneh, Henok Legesse, Yalew Mossie, Tilahun Teshager, Eyerusalem Tamiru, Diribsa Tizau, Tesfaye Asfaw

**Affiliations:** ^1^ Department of Pediatrics and Child Health Nursing, School of Nursing, College of Health and Medical Sciences, Haramaya University, Harar, Ethiopia; ^2^ Department of Emergency and Critical Care Nursing, School of Nursing, College of Health and Medical Sciences, Haramaya University, Harar, Ethiopia; ^3^ Department of Comprehensive Nursing, School of Nursing, College of Health and Medical Sciences, Haramaya University, Harar, Ethiopia

**Keywords:** parental, traditional medicine, children, Harar, Ethiopia

## Abstract

**Introduction:**

Globally, there is consensus that traditional medicine (TM) has its benefit in solving health-related issues; however, a challenge lies in the lack of standardized scientific criteria to guide its appropriate use. Evidence suggests that, in some cases, traditional medicine may exacerbate health problems and lead to complications. Although many studies have explored traditional medicine use among adults, there is limited research on parental use of traditional medicine for children, particularly in Harar City. Therefore, the aim of this study was to determine the prevalence and associated factors of parental traditional medicine use for children in Harar City, Eastern Ethiopia.

**Research methods:**

A cross-sectional study was carried out in the community of Harar City between 15 October and 15 November 2024. A multistage sampling procedure was used to obtain 348 parents. The required data were assembled through in-person interviews. The data that assembled were analyzed using Stata statistical software, version 16.0. To determine the associated factors with the outcome variable, multivariable logistic regression was applied via adjusted odds ratio (aOR) with a 95% confidence interval (CI). Independent variables with *p* < 0.05 were declared as significantly associated variables with the outcome variable.

**Results:**

Approximately 348 parents were sampled for the study with a response rate of 100%. The proportion of parental TM use for children was 76.15% (95% CI: 71.8–80.5). Parents with more than four children (aOR = 3.24, 95% CI: 1.13–9.28), with a good attitude toward TM (aOR = 1.98, 95% CI: 1.02–3.86), and who were members of community-based health insurance (aOR = 0.34, 95% CI: 0.19–0.63) were independent variables associated with parental traditional medicine use for children.

**Conclusion:**

Approximately three-quarters of parents reported using traditional medicine for their children. This practice was influenced by modifiable factors. Therefore, stakeholders in the health sector should give focused attention to the key factors influencing parental use of traditional medicine for children.

## Introduction

Traditional medicine (TM) encompasses the therapeutic practices rooted in traditional knowledge and culturally specific beliefs that have been developed over generations within various societies, including indigenous communities, before the advent of modern science (1, 2). It refers to the knowledge, skills, and practices based on the theories, beliefs, and experiences indigenous to different cultures—whether scientifically explained or not—used for maintaining health as well as for the prevention, diagnosis, and treatment of physical and mental illnesses (3, 4).

In the previous decade, the development and mass production of chemically synthesized drugs have revolutionized healthcare in most parts of the world (5). However, large sections of the population in developing countries still depend on traditional medicine for their primary healthcare needs (6). The global disease burden is growing rapidly; as a result, indigenous African medicine offers an alternative and affordable remedy that is accessible to millions who cannot afford or access modern healthcare due to its high cost and limited availability (7, 8).

Various forms of traditional medicine are practiced across different cultures, reflecting their diversity. These include bone setting, puncture-induced bleeding, cupping, cauterization, scarification, tooth extraction (9), use of mineral substances, herbal remedies, animal products, and spiritual therapies like *tsebel* (holy water). Manual techniques are also applied, either alone or in combination (10), through non-invasive or invasive procedures (11). People depend on traditional medicine because it is easily accessible, affordable, and low cost, particularly in contexts marked by poverty, illiteracy, and limited access to modern healthcare services (12, 13).

Globally, approximately 80% of the population relies on TM for their primary healthcare needs, with most remedies derived from natural products, animals, and plants (14, 15). In developing countries, specifically in Asia and Africa, the use of TM is in the range of 70%–95% (16). In Africa, especially sub-Saharan countries, approximately 90% of the population depends on TM (17, 18). In Ethiopia, more than 80% of the population depends on TM for their primary healthcare needs (19). Approximately 71%–90.3% of Ethiopian parents have used different types and forms of TM for their children (20). The most commonly practiced traditional therapies include herbal remedies (66.9%), religious treatments (52.8%), massage (22.8%), bone setting (21.8%), and cutaneous cauterization (43.6%) (20, 21).

Even though traditional medicine offers benefits for human health, a major concern lies in the lack of scientifically proven standards—particularly regarding appropriate usage, dosage, and route of administration (22). Studies have shown that some traditional medicine therapies used in children by their parents have had adverse outcomes (23). More than half of all TMs are prescribed, dispensed, and sold illegally by traditional healers and approximately half of all users use TM incorrectly (24).

Many patients are hospitalized due to the adverse effect of and complications associated with TM (21, 25). The secretive nature of some traditional healers creates barriers to effective treatment and prevention of such complications (26). Invasive traditional herbal medicines result in a wide range of complications, from mild allergic reactions and gastrointestinal issues to severe outcomes such as respiratory problems, blood clotting disorders, blood pressure issues, liver toxicity, renal failure, and multi-organ dysfunction syndrome (27). Among children who receive invasive traditional herbal medicine, approximately 30.96% develop multi-organ dysfunction syndrome (28). In addition, the most commonly affected organs include the kidney (renal failure, 17.74%), nervous system (neurological failure, 15.16%), and heart (heart failure, 14.52%), with nearly one-third of them dying in intensive care units (29). Over 25% of children admitted to the pediatric intensive care units were suspected of experiencing toxicity due to traditional herbal medicine use (30, 31).

Traditional cutaneous cauterization results in a wide variety of complications, including wound infection, abscess, septic shock, deep skin burn, disfigurement from contractures, scars, hair loss, keloids, carcinoma, oral complications, aspiration, edema, and viral transmission, including hepatitis, HIV, and tetanus (32, 33).

Children, being dependent on their parents, are more vulnerable to TM, which can compromise their health rights and hinder progress toward achieving Millennium Development Goal 4 (MDG4) (34, 35). Providing evidence-based information on parental use of TM in children is crucial. This can inform the development of appropriate control measures to ensure the quality and safety of these practices. As a result, this will help improve child health outcomes and reduce TM-related morbidity and mortality among children (36).

There have been many studies conducted on adult TM use; however, there are limited studies conducted on parental use of TM for children, particularly in Harar City. Therefore, the objective of this study was to determine the prevalence and associated factors of parental traditional medicine use for children in Harar City, Eastern Ethiopia.

## Research methods and materials

### Study design, area, and period

A cross-sectional study was carried out in the community of Harar City, Harari regional state, in the eastern part of Ethiopia. Harar is the principal city of Harari regional state and is one of the most ancient and historic cities of Ethiopia, located approximately 526 km away from the capital city of Addis Ababa. The Harari region is entirely surrounded by the neighboring Oromia region. According to the 2007 report from the National Central Statistical Agency, Harari region had a total population of 183,415, with 92,316 men and 91,099 women. Regarding health facilities in the region, there are 6 hospitals (3 governmental, 2 private, and 1 non-governmental or fistula center), 8 health centers, 29 private clinics, 26 health posts, and 1 regional laboratory. These facilities are supposed to provide health services to the population in this region. These data were obtained from the Harari Regional Health Bureau. Harar City is divided into 9 woredas and 26 localities (kebeles). The study was conducted between 15 October and 15 November 15 2024.

### Source and study population

In this study, the population source include all parents with children who live in Harar City. The study population in this research include all parents with children live in randomly chosen kebeles.

### Inclusion and exclusion criteria

All parents with children who have lived for at least 6 months in randomly selected kebeles were included in the study. Parents who were seriously ill while data were being gathered were excluded.

### Sample size calculation

The sample size for this study was calculated using Epi info statistical software version 7.2.6.0, based on several assumptions. These included 95% confidence interval (CI), 80% power, and an odds ratio (OR) of 2.49 for a variable significantly associated with the outcome in a previous study. The proportion of the outcome in the unexposed group was assumed to be 32%, with an unexposed-to-exposed ratio of 1:1, as the study used a cross-sectional design. In addition, a 10% non-response rate was factored in, and the resulting was multiplied by a design effect of 2 to account for the sampling procedure. The final calculated sample size for the study was 348 participants.

### Sampling techniques/procedure

**Figure 1:**
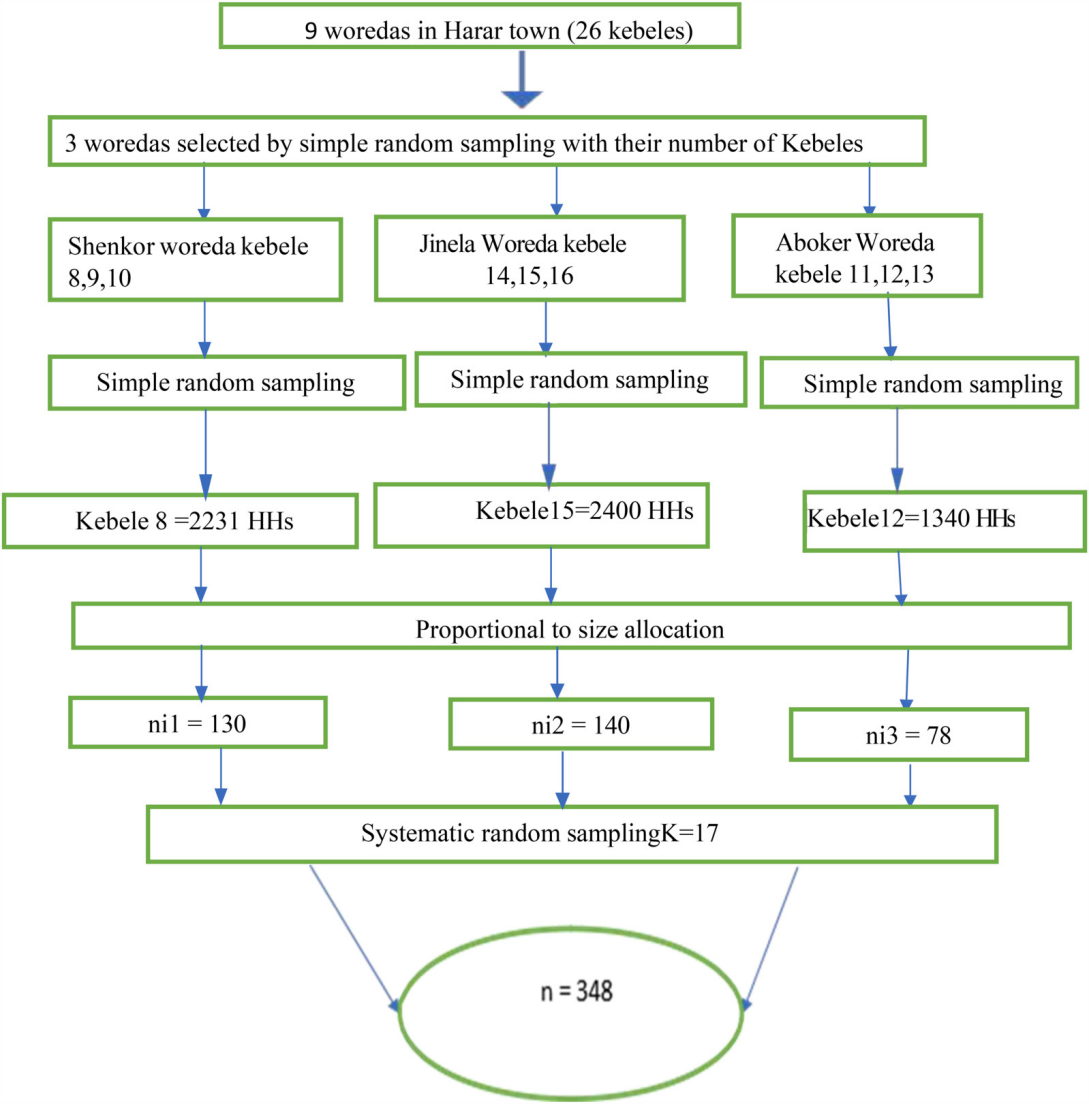
Multistage sampling procedure to recruit study participants from the source population.

In the present study, a multistage sampling technique was employed to recruit participants. Harar City is composed of 9 woredas and 26 kebeles. Initially, three woredas were selected using the lottery method. From each of the selected woredas, one kebele was randomly chosen, also using the lottery method. The total sample size of 348 participants was then allocated proportionally to the population size of each selected kebele. Thereafter, respondents from each selected kebele were chosen using a systematic random sampling technique. The first respondent was chosen at random, and subsequent participants were selected at regular intervals thereafter (Figure 1).

### Data collection method

A structured questionnaire with in-person interviews was used to gather the required data from the study participants. The questionnaire used in this study was modified into our research context from preceding research works that were conducted on similar topics in Ethiopia. The questionnaire consisted of sociodemographic characteristics of the parents, traditional medicine use by parents for children and themselves, the attitude of parents toward TM, factors related to parental healthcare experience, factors that enabled parents to use TM, and need factors. The data collection tool (questionnaire) was primarily prepared in the English language; we then translated it into the local languages of the community (Affan Oromo and Amharic) and back into English by another person who was fluent in both languages to check for consistency and equivalency. Five Bachelor of Science (BSc) nurses were the data collectors and three Master of Science (MSc) graduates were the supervisors in the data collection process.

### Data quality control

Before initiating the data collection process, the data collection tool (questionnaire) was pretested in a similar setting outside the study area to ensure the authenticity and quality of the collected data. Based on pretest findings, necessary corrections were made, and the final version of the corrected questionnaire was approved for use. The internal consistency of the tool was checked using reliability analysis, yielding a Cronbach's alpha value of 0.82, which falls within the acceptable range. Both data collectors and supervisors were thoroughly trained on the entire data collection process before commencement. During data collection, trained supervisors closely monitored the process to ensure coherence and completeness. During data management, storage, and analysis, the collected data were extensively checked for completeness and coherence.

### Definitions

The term “parent” in our study refers to the mother, father, or legal guardian who cares for the child.

The term “child” refers to individuals aged under 18 years.

Traditional medicine use refers to anything that is not considered a part of contemporary modern medicine (MM) even though it is acknowledged in the community and the community used it for the treatment and prevention of illness as well as to promote health. Traditional medicine is neither ordered by the healthcare provider nor utilized normally as a diet in that specific community.

In this study traditional healers are individuals providing healthcare without being trained in modern medical science.

A bone settler is a traditional practitioner who has no training in modern medical science but provides therapy for bone dislocations or fractures, such as balancing the dislocated bone and helping in the manipulation of joints and muscles.

The term “herbal medicine” refers to culturally acceptable traditional medicines that are prepared from plants to cure illness.

Religious therapy is the treatment and prevention of illness as well as health promotion with the help of the healing presence of God or Allah.

A massage is the relief of pain and tension by placing mild or strong pressure on the joints and muscles of a person and applied by a locally known traditional healer.

### Data processing and analysis

After the data collection process was finalized, the data were entered into Epi data statistical software version 4.6 and exported to Stata version 16 statistical software for coding, editing, cleaning, and analyzing.

To describe continuous variables, descriptive statistics such as means and standard deviations (SDs) were used. Categorical variables were described using frequencies and percentages. To identify factors associated with parental TM use, a bi-variable logistic regression analysis was conducted. Initially, bi-variable logistic regression analysis was conducted to screen potential candidate variables. Independent variables with *p* ≤ 0.25 were then included in the multivariable logistic regression analysis to avoid possible confounding effects.

The association between the independent variables and the outcome variable was assessed using a multivariable logistic regression analysis. The model's goodness of fit was evaluated using the Hosmer and Lemeshow test (*p* = 0.56). To check for multicollinearity, a variance inflation factor (VIF) analysis was performed, and no evidence of multicollinearity was found. Independent variables with *p* < 0.05 and adjusted odds ratio (aOR) with 95% confidence intervals were considered statistically significant. The results were presented in the form of text, figures, and tables.

### Ethical consideration

Ethical clearance for this study was obtained from the Institutional Health Research Ethics Review Committee of Haramaya University, College of Health and Medical Sciences (reference no. IHRERC/286/2024). A formal letter of cooperation was submitted to the Harari Region Health Bureau. Before data collection, written informed consent was obtained from all participants. Participation was voluntary, and each participant was assured that their responses during the in-person interview would remain strictly confidential.

## Results

### Sociodemographic characteristics

A total of 348 parents were sampled for the study, with a response rate of 100%. In our study, more than half (*n* = 217, 62.40%) of the study participants were women. The mean age of the participants was 33 ± 9.4 years. Regarding the religion of the study participants, 172 (49.40%) were followers of the Orthodox religion. The majority of the participants (*n* = 323, 92.80%) were married. Regarding the educational status of the respondents, 52 (14.90%) participants could not read and write. Of the participants, 125 (35.90%) listed their occupation as housewife. Most of the parents (*n* = 144, 41.40%) have three to four children (mean 3.15 ± 1.5). In our findings, the majority of the participants (*n* = 237, 68.10%) had a monthly income of more than 2,000 Ethiopian Birr (ETB) (Table 1).

**Table 1:** Sociodemographic characteristics of parents in Harar City, Eastern Ethiopia, 2024 (*n* = 348).

Variable	Response	Frequency	Percent
Sex of the parents	Male	131	37.60
	Female	217	62.40
Age of parents (years)	≤20	9	2.60
	21–30	120	34.50
	31–40	126	36.20
	41–50	68	19.50
	>50	25	7.20
Religion of the parents	Orthodox	172	49.40
	Muslim	141	40.50
	Protestant	32	9.20
	Catholic	3	0.90
Marital status of the parents	Single	11	3.20
	Married	323	92.80
	Divorced	9	2.60
	Widowed	5	1.40
Educational status of the parents	Cannot read and write	52	14.90
	Can read and write	100	28.70
	Primary school (1–8)	117	33.60
	High school (9–12) and above	79	22.70
Occupation of the parents	Housewife	125	35.90
	Government employee	91	26.10
	Merchant	97	27.90
	Non-governmental organization	20	5.70
	Daily laborer	10	2.90
	Farmer	5	1.40
Monthly income of household	≤2,000	111	31.90
	2,001–10,000	209	60.10
	>10,000	28	8.00
Number of children under 18 years	1–2	135	38.80
	3–4	144	41.40
	>4	69	19.80

### Proportion of parental traditional medicine used for children

The proportion of parental TM use for children was 76.15% (95% CI: 71.8–80.5)**.** Regarding the types of TM used by parents for their children, the majority (*n* = 154, 58.10%) had applied herbal treatments. Regarding the type of TM utilized for children by their parents, most of the parents (*n* = 168, 63.40%) used a drinkable form of TM (Table 2).

**Table 2:** Parental use of traditional medicine for children in Harar City, Eastern Ethiopia, 2024 (*n* = 348).

Variable	Response	Frequency	Percent
Parental traditional medicine use for children	Yes	265	76.15
	No	83	23.85
Time of TM use for child in the last 12 months	Within the 1 month	121	45.70
	Within the 6 months	71	26.80
	Before 6 months	73	27.50
What types of TM you used for your children	Religious/prayer	69	26.00
	Herbal medicine	154	58.10
	Bone settler	18	6.80
	Massage	24	9.10
Form of TM used	Drinkable form	168	63.40
	Ingestible form	49	18.50
	Ointment form	36	13.60
	Tsifet	12	4.50
Why not used TM for your children	Fear of side effect	37	44.60
	Access to MM	26	31.30
	Difficultly in accessing TM	2	2.40
	Religion	9	10.80
	Not curable	9	10.80

### Parental enabling and need factors to use TM for children

We asked the study participants to tell us the main reasons for using TM for their children. Of them, 108 (40.80%) responded that TM was easily accessible in their environment. Similarly, the participants were asked about the source of information for using TM for their children; of them, 116 (43.80%) replied that a family member was the source of information. Most of the parents (*n* = 157, 59.20%) used TM for their children as a disease treatment. Of the parents, 64 (24.20%) used TM as a treatment for respiratory disease and 43 (16.20%) used TM to treat gastrointestinal problems (Table 3).

**Table 3:** Enabling and need factors for parental use of traditional medicine for children in Harar City, Eastern Ethiopia, 2024 (*n* = 265).

Variable	Response	Frequency	Percent
Reasons for applying to TM for children	Easily accessible of TM	108	40.80
	Cheap in price	68	25.70
	Having low income	47	17.70
	Referred by someone	30	11.30
	Inaccessibility of healthcare	12	4.50
Sources of information about TM use for children	Self	50	18.90
	Family	116	43.80
	Relative	23	8.70
	Friends	34	12.80
	Health professionals	2	0.80
	Religious institutions	9	3.40
	Traditional healers	19	7.20
	Media	10	3.80
	Neighbors	2	0.80
Purpose of the parent to use TM for children	To promote health	51	19.20
	To prevent illness	41	15.50
	To treat illness	157	59.20
	To prevent deformity	16	6.00
Overall health status of the child after TM used	Very poor	8	3.00
	Poor	10	3.80
	Fair	48	18.10
	Good	95	35.80
	Very good	104	39.20
Illness that parents frequently used TM for children	Pulmonary	64	24.20
	Gastrointestinal	43	16.20
	Urological	18	6.80
	Psychosomatic	22	8.30
	Dermatological	30	11.30
	Musculoskeletal	27	10.20
	Common cold and evil eye	29	10.90
	Endocrine	17	6.40
	Chronic fatigue	15	5.70
Duration of illness	Acute (<6 months)	98	37.00
	Chronic (≥6 months)	167	63.00

### Parental healthcare experience and their attitudes toward TM

We asked the parents whether they used TM for themselves and learned that 165 (47.40%) were self-TM users. Among those users, 111 (67.30%) were women (mothers). The study participants were requested to rate their level of satisfaction with TM, with 36 (21.80%) replying they were satisfied. In total, 158 (45.40%) participants replied that TM has good efficacy. Of the participants, 230 (66.10%) had a good attitude toward TM. More than half of the participants (*n* = 261, 75.00%) had future plans to use TM. A total of 238 (68.40%) study participants encouraged others to use TM. Approximately 218 (62.60%) participants said that modern medicine cannot cure certain diseases. In our study findings, half of the participants were members of the community-based health insurance (CBHI) plan (Table 4).

**Table 4:** Parental healthcare experience and attitudes toward use of traditional medicine for children in Harar City, Eastern Ethiopia, 2024 (*n* = 348).

Variable	Response	Frequency	Percent
Parental TM use for themselves	Yes	165	47.40
	No	183	52.60
Parents who used TM	Mother only	111	67.30
	Father only	51	30.90
	Both	2	1.20
	Guardian	1	0.60
Parental reason for applying TM than MM	When selected correctly it is effective	35	21.20
	Satisfaction with TM	36	21.80
	Dissatisfaction of MM	24	14.50
	Fear of using drugs side effects	13	7.90
	Difficulty in accessing healthcare facilities	8	4.80
	Less efficacy of MM	10	6.10
	No cure/prevent by MM	26	15.80
	Religion	13	7.90
How to indicate level of satisfaction after TM used?	Completely dissatisfied	14	8.50
	Somewhat dissatisfied	17	10.30
	Neither satisfied nor dissatisfied	8	4.80
	Somewhat satisfied	58	35.20
	Completely satisfied	68	41.20
Parental experience with modern healthcare system	Bad	21	6.00
	Medium	144	41.40
	Good	183	52.60
Parental attitudes toward TM use	Good attitude	230	66.10
	Poor attitude	118	33.90
Parents who had plan to use TM in the future	Yes	261	75.00
	No	87	25.00
Parents agree with usage of TM among the community	Strongly agree	73	21.00
	Agree	158	45.40
	Neutral	63	18.10
	Disagree	30	8.60
	Strongly disagree	24	6.90
Parents encourage others to use TM	Yes	238	68.40
	No	110	31.60
Diseases not cured by MM	Yes	218	62.60
	No	130	37.40
Member of CBHI	Yes	174	50.00
	No	174	50.00

### Parental use of traditional medicine for children and associated factors

The results of the multivariable logistic regression analysis revealed that the number of children, parental attitudes toward TM, and membership in the community-based health insurance plan were significantly associated with parental use of traditional medicine for children. Parents with more than four children were three times more likely to use TM for their children compared to those with less than four children (aOR = 3.24, 95% CI: 1.13–9.28). Similarly, parents who had a positive attitude toward TM were twice as likely to use it for their children compared to those with a negative attitude (aOR = 1.98, 95% CI: 1.02–3.86). Finally, CBHI was also significantly associated with TM use; parents who were members of CBHI were 66% less likely to use TM for their children compared to non-members (aOR = 0.34, 95% CI: 0.19–0.63) (Table 5).

**Table 5:** Factors associated with parental use of TM for children in Harar City, Eastern Ethiopia, 2024 (*n* = 348).

Variable	Response	Parental TM use for children		cOR (95% CI)	aOR (95% CI)	*p*-value
		No	Yes			
Sex	Male	26	105	1	1	
	Female	57	160	1.44 (0.85–2.43)*	2.03 (0.96–4.29)	0.065
Age	≤20	4	5	1	1	
	21–30	41	79	1.54 (0.39–6.05)	0.77 (0.17–3.57)	0.74
	31–40	16	110	5.50 (1.34–22.7)*	2.47 (0.47–12.86)	0.28
	41–50	17	51	2.40 (0.58–9.97)*	1.11 (0.21–5.81)	0.89
	>50	5	20	3.20 (0.62–16.5)*	1.69 (0.28–10.33)	0.57
Religion	Orthodox	47	125	0.75 (0.30–1.84)		
	Muslim	27	114	1.18 (0.46–3.02)		
	Catholic	2	1	0.14 (0.01–1.78)		
	Protestant	7	25	1		
Marital status	Single	2	9	1		
	Married	77	246	0.71 (0.15–3.36)		
	Divorced	2	7	0.78 (0.09–6.98)		
	Widowed	2	3	0.33 (0.03–3.52)		
Education	Cannot read and write	10	42	0.83 (0.33–2.06)	1	
	Can read and write	31	69	0.44 (0.21–0.91)*	0.71 (0.25–1.96)	0.5
	Primary school	29	88	0.59 (0.29–1.24)*	0.49 (0.19–1.28)	0.15
	High school and above	13	66	1	0.69 (0.27–1.74)	0.43
Occupation	House wife	39	86	0.55 (0.11–2.72)		
	Farmer	1	4	1.00 (0.07–14.6)		
	NGO	3	17	1.42 (0.19–10.23)		
	Government employee	21	70	0.83 (0.16–4.23)		
	Merchant	17	80	1.18 (0.23–6.04)		
	Daily laborer	2	8	1		
House hold monthly income	≤2,000	18	93	2.87 (1.14–7.23)*	0.68 (0.34–1.4)	0.28
	2,001–10,000	55	154	1.56 (0.68–3.58)	0.50 (0.17–1.52)	0.22
	>10,000	10	18	1	1	
Number of children below 18	1–2	42	93	1	1	
	3–4	35	109	1.41 (0.83–2.38)*	1.28 (0.68–2.42)	0.44
	>4	6	63	4.74 (1.9–11.82)*	3.24 (1.13–9.28)	0.028*
Self-TM use	Yes	31	134	1.72 (1.04–2.85)*	0.96 (0.50–1.85)	0.91
	No	52	131	1	1	
Attitude	Good attitude	41	189	2.55 (1.54–4.23)*	1.98 (1.02–3.86)	0.045*
	Poor attitude	42	76	1	1	
Member of CBHI	Yes	60	114	0.29 (0.17–0.49)*	0.34 (0.19–0.63)	0.001*
	No	23	151	1	1	

TM, traditional medicine; cOR, crude odds ratio; CI, confidence interval; aOR, adjusted odds ratio; NGO, non-governmental organization.

* *p*-value <0.05 (significance).

## Discussion

Traditional medicine practice has its own benefit in solving health-related problems in society; however, there is a problem related to the lack of scientifically proven standards for deciding its appropriate use, including the dose of medicine and route of administration (37). Studies have shown that most patients who used traditional medicine experienced a worsening of their condition and developed new complications (38). Identifying risk factors and implementing safety measures are vital to reducing child morbidity and mortality related to traditional medicine use. The objective of this research was to assess the proportion of parental TM use for their children and its associated factors in Harar City, Eastern Ethiopia.

In our study, the proportion of parental TM use for their children was 76.15% (95% CI: 71.8–80.5). The multivariable analysis identified the number of children parents had, their attitude toward TM, and membership in CBHI as factors associated with parental traditional medicine use for children.

The results of this study were almost consistent with those of previous studies conducted in Fagita Lekoma woreda, Ethiopia (71.0%) (20). Similarly, the findings of this study were almost aligned with the study conducted in the Oromia region of Ethiopia (79.4%) (39).

On the other hand, the results of this study were lower than findings in previous studies conducted in Motta Town, Ethiopia (88.2%) (11), Axum Town (87.8%) (40), North Mecha District (90.3%) (19), and East Wollega (94.2%) (41). This lower prevalence of parental TM use for children in the study area might be due to differences in the availability of modern health facilities or their distance from the villages. In areas where modern healthcare facilities are scarce or located far from the community, the use of TM tends to increase (42). Another possible explanation for this difference may be variations in how much attention is given to child health by health sector stakeholders. This includes differences in public health education, awareness campaigns, and the commitment of community health extension workers to educate communities about the adverse complications of TM use (43). In this regard, since Harai Regional State is the smallest in Ethiopia in terms of both land mass and population, health sector stakeholders may have had a better opportunity to raise awareness about the risks of using unproven traditional medicine. Consequently, TM use may be lower in this area. Lastly, differences in cultural and religious beliefs regarding TM use across regions may also contribute to the variation (44, 45).

In the same fashion, this study's result was lower than findings from studies conducted in Shendi Town, Sudan (92%) (46), Nigeria (84%) (47), and Ghana (86.1%) (12). This discrepancy might be due to cultural, behavioral, health policy, and monitoring differences across countries.

However, the results of the present study were higher than those reported in Korea (65.3%) (48), Australia (68.5%) (49), the UK (38%) (50), and Europe (52%) (51). The higher prevalence of TM use observed in our study may be due to limited access to modern healthcare facilities in the area (52, 53), as well as historical and sociocultural ties to TM (54, 55). Another possible reason could be the lower cost of TM compared to modern treatments, its ease of accessibility in the local environment, and the high price of modern medicines. In addition, differences in health policies and controlling measures surrounding TM use may also be reasons (56–58). Moreover, the high proportion of TM use might be linked to the unavailability of scientifically proven modern treatments for certain conditions, including both non-communicable and communicable diseases (59–61).

Regarding the factors influencing parental use of TM for their children, this study found that several variables played a role. In our findings, there were increased odds of using TM for children among parents who had more than four children compared with those with fewer than four. This finding is supported by a study conducted in Uganda (56). The possible explanation for this association is that parents with larger families may be unable to afford the medical fees associated with modern healthcare services (62). In contrast, TM is often more accessible at lower costs. In addition, traditional healers may accept alternative forms of payment, such as goods, labor, or credit (63). When treating personalistic illnesses, traditional healers often involve the extended kin group, and there may be strong social pressure from family elders to comply with traditional practice, which can include providing financial support (63). On the other hand, in modern health facilities, the medical fees are typically covered by the patient or other relatives (64).

Parental attitude toward traditional medicine use in children plays a key role in determining its utilization. In this study, parents with a positive attitude toward TM were more likely to use it for their children compared to those with a negative attitude. This finding is supported by studies conducted in Plateau State, Nigeria (13), in the University of Karbala, Iraq (65), East Wollega Zone (41), and Mecha District, Ethiopia (19). One possible explanation is that parents who think TM is a preferred form of healthcare are more inclined to use it over modern medicine (15).

Another enabling factor that that affects parental use of traditional medicine in children is membership in CBHI. Participants who were being members of community-based health insurance were less likely to use traditional medicine for their children compared to those who were not members. This finding is consistent with studies conducted in Ethiopia and Tanzania (20, 66). An explanation is that CBHI helps solve financial barriers to accessing modern medical services (67).

## Study limitations

In this study, we applied a cross-sectional design, which may not establish a cause-and-effect relationship when assessing factors associated with the outcome variable.

## Conclusion

Approximately three-quarters of parents reported using TM for their children. This practice was influenced by modifiable factors. Therefore, stakeholders in the health sector should give specific attention to these influencing factors to address the use of traditional medicine in children more effectively.

## Data Availability

The raw data supporting the conclusions of this article will be made available by the authors, without undue reservation.
